# Discordance in postnatal care between mothers and newborns: Measurement artifact or missed opportunity?

**DOI:** 10.7189/jogh.10.010505

**Published:** 2020-06

**Authors:** Agbessi Amouzou, Elizabeth Hazel, Lara Vaz, Sanni Yaya, Allisyn Moran

**Affiliations:** 1 Department of International Health, Johns Hopkins Bloomberg School of Public Health, Baltimore, Maryland, USA; 2 Save the Children, Washington, D.C., USA; 3 School of International Development and Global Studies, University of Ottawa, Ottawa, Ontario, Canada; 4 World Health Organization, Geneva, Switzerland

## Abstract

**Background:**

Postnatal care (PNC) for mothers and newborns is essential to monitor risks of morbidity and adverse conditions following delivery. Current estimates of the coverage of PNC show substantial discordance between mothers and newborns. We investigate the sources of this discordance in Demographic and Health Surveys (DHS).

**Methods:**

We used DHS data from 48 countries collected since 2011, spanning phases 6 and 7 of the survey program with 32 and 16 surveys, respectively, analyzed. We assessed the distribution of the reported timing of PNC and conducted a sensitivity analysis that excludes/includes PNC reported within 0-1 hour or PNC in the day 2. Agreement in PNC reporting considered four groups: (1) Concordance, neither mother nor newborn received PNC; (2) Concordance, mother and newborn pair received PNC; (3) Discordance, mother received PNC and newborn did not; of (4) Discordance, mother did not receive PNC but the newborn did. We carried out logistic regressions to understand correlates of PNC discordance. All analyses distinguished phase 6 surveys from phase 7.

**Results:**

We found substantial differences in the PNC coverage estimated between phase 6 and phase 7 surveys. The phase 7 PNC questions for newborns were improved to increase the understanding of the questions by respondent which probably led to reducing the large PNC gap between mothers and newborns observed in phase 6 surveys. With phase 6 surveys, PNC coverage for mother was estimated on average at 62% compared to only 31% for newborns. No such gap was observed for phase 7 surveys, where for both mothers and newborns, the PNC coverage estimate was similar, at 56%. For both phases, over half of the reported PNC for mothers and newborns occurred during 0-1 hour following delivery, leading to substantial overestimation of PNC coverage, due to confusion between intrapartum care and PNC. There were 37% discordant cases between mother and newborn, largely in favor of the mother in phase 6 surveys, compared to 16% in phase 7 surveys. In phase 6 surveys, discordant PNC cases were observed largely among facility deliveries vs non-facility deliveries (44% compared to 19%).

**Conclusions:**

Current estimates of coverage of PNC from DHS phase 6 surveys appears to include substantial level of measurement noises that could explain substantial part of the mother-newborn discordance in PNC. The PNC estimates appear to capture a substantial number of intrapartum care. Current measurement approaches warrant further validation to ensure accurate monitoring of the PNC programs.

Postnatal care for mothers and newborns is a critical package of interventions to monitor the health status of mothers and newborns during the six weeks following delivery. It has been shown to be effective in reducing neonatal mortality [1-3]. The 2013 WHO guidelines prescribe integrated postnatal care for the both the mother and the newborn, with the first health check occurring within the first 24 hours after birth. Three additional health checks for the mother and newborn are recommended successively on day three, between day seven and fourteen, and six weeks after birth. For facility deliveries, care must be provided in the facility for at least 24 hours [4]. The recommended content for newborn postnatal care includes an assessment of the newborn for any abnormal signs, exclusive breastfeeding, and cord care. For the mother, a complete assessment, complemented with iron and folic acid supplementation, counseling and psychosocial support are recommended. Postnatal care is distinct from immediate newborn care which includes resuscitation, thermal care and breastfeeding initiation. For the mother, providers check for signs of hemorrhage and infection during the 24 hours and continue monitoring health throughout the postnatal period and is distinct from intrapartum care [5].

Measuring the coverage of postnatal care contacts in household surveys has been challenging; Moran et al discussed extensively the measurement challenges of postnatal care for both the woman and the newborn [6]. To date, only a postnatal care contact indicator has been defined at global level and adopted by countries. The global indicator defines postnatal care contact as health check received within two days of delivery. The two largest household survey programs in low- and middle-income countries, the Demographic and Health Surveys (DHS) and the Multiple Indicator Cluster Surveys (MICS), have developed approaches to measure this indicator. Although efforts have been made by the two survey programs to harmonize their approaches, they continue to implement slightly different algorithms for measurement, which makes comparability difficult [7].

Furthermore, the assessment of postnatal care contacts indicates substantial level of discrepancy in the report of care received by the mother and the newborn. The Countdown to 2030, which monitors coverage of effective interventions in 81 countries in LMIC, reported median postnatal care for mothers at 59% compared to 42% for babies for the period 2012-2016 [8]. Such a result is surprising given the recommendation for integrated postnatal care for both mother and newborn. The discrepancy may be indicative of the quality of postnatal care service provision. It can also originate from misreporting from mothers based on their understanding of the questions, their recall of postnatal services received, and whether mother and newborn have been separated at some point following the delivery, leading the mother to be unaware of any care provided to the newborn, including the possibility that only one of the dyad is actually receiving PNC.

The discordance in PNC for mother and newborn has not been studied in the literature. This paper assesses this discordance in low and middle-income countries with available recent DHS surveys. More specifically, we quantify the gap in coverage of PNC for mothers and newborns, the level of discordance and concordance in reporting of PNC between mothers and newborn within individual dyads. We assess the reported timing of PNC and carry out sensitivity analysis to understand whether there is possible confusion between intrapartum care and postnatal care, the possibility of differential recall by respondent during the household survey, and the effect it has on coverage estimates. Finally, we analyze socio-demographic, economic and service provision factors associated with the likelihood of discordance in reported receipt of PNC.

## DATA AND METHODS

We used available data from Demographic and Health Surveys (DHS) carried out since 2011. DHS is a nationally representative sample survey carried out generally about every five years in most low and middle-income countries. The survey interviews head of households, all women aged 15-49 and men aged 15-59 (or 15-64 in some cases). Women’s interviews include information on their complete birth history. For women who had a birth in the past five years, additional health-related questions, including maternal and newborn care questions, are posed. We retained most recent surveys which included questions measuring postnatal care of both woman and newborn and restricted the analysis to births in the two years preceding the survey. Data were downloaded from the DHS website (dhsprogram.com) as of 4^th^ April 2019. A total of 48 countries are included in the analysis, representing 280 651 births in the two years preceding the surveys. This includes 32 surveys from phase 6 (accounting for 223 500 births) and 16 surveys from phase 7 (accounting for 57 151 births). Table S1 in the **Online Supplementary Document** includes the list of countries included in the analysis.

### The PNC questions in DHS

**Figure 1:**
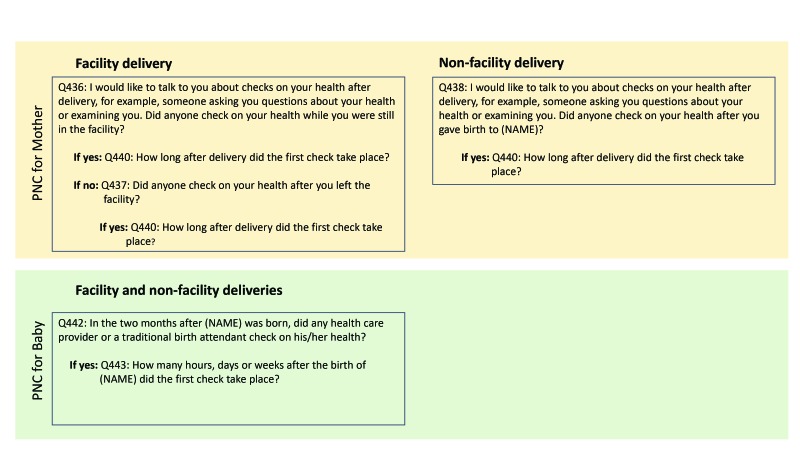
Demographic and Health Survey (DHS) 6 design for postnatal care (PNC) questions for mothers and newborns.

**Figure 2:**
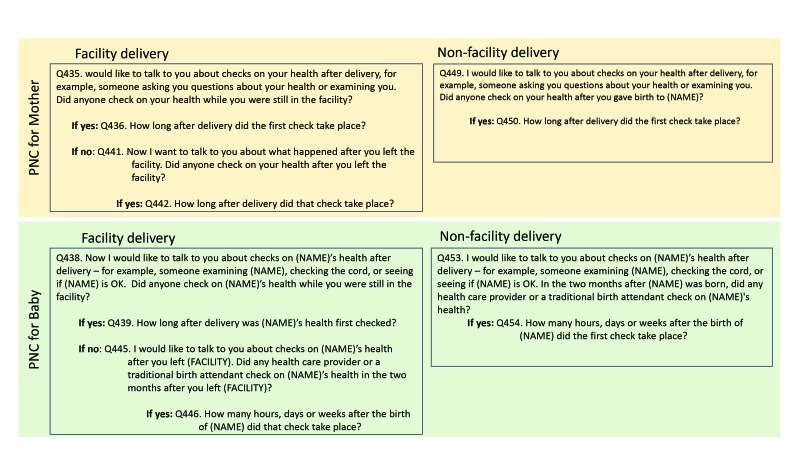
Demographic and Health Survey (DHS) 7 design for postnatal care (PNC) questions for mothers and newborns.

The DHS program revises its questionnaire according to phases of five years. Data since 2011 span phases 6 (DHS6) and 7 (DHS7). The questionnaire module for newborn PNC was expanded starting in phase 7. **Figure 1** and **Figure 2** show the questions asked to elicit PNC information in the respective survey phases. There were differences in the wording and structure of the questions between the two phases. For mothers, the set of questions were similar between the two phases regardless of place of birth. For newborns, DHS7 distinguished between facility vs non-facility births and asked a separate set of questions for each; DHS6 on the other hand, had a single question regardless of place of birth. Furthermore, a two-month window was included in the newborn PNC questions in DHS6; in DHS7, this window was referenced only for non-facility births and facility births for whom the mother reported that no health check was performed on the newborn before discharge. Questionnaire differences between DHS6 and DHS7 are a potential source of measurement differences in the PNC coverage estimates.

### The PNC indicator

While the WHO guidelines recommend several PNC contacts with specific timing, for measurement and monitoring, the global community has focused mainly on the first contact. The global PNC contact indicator is defined as PNC intervention received within 48 hours of delivery, regardless of the place of delivery [5]. The PNC indicator captures contact with a health provider and the timing of the contact. For both mothers and newborns, we calculated the global indicator, restricting to deliveries in the past two years preceding the survey to reduce potential effects of recall.

### Statistical analysis

We carried out descriptive analysis of PNC indicators and a multilevel logit regression of PNC discordance. Given the 48 hours window following the birth that is used to define the global PNC indicator, a major constraint affecting the indicator is the ability to distinguish intrapartum and essential newborn care provided immediately following delivery to mothers and newborns from actual postnatal care. To understand this possible overlap, we first analyze the timing of the reported first PNC contact to capture its distribution. We then carried out sensitivity analyses of the global PNC indicator by including/excluding the first PNC reported to have occurred within 0 or 1 hour. Similarly, there is usually a reporting confusion between day 0 and day 1 making it possible that day 2 is counted as part of the 48 hours following delivery. We therefore also compared the PNC contact indicator, including/excluding day 2. We carried out the analyses by country and also for the pooled country data sets.

To analyze the PNC contact discordance between mother and newborn we distinguished four categories: (1) negative concordance with neither mother nor newborn received PNC; (2) positive concordance when both mother and newborn pair received PNC; (3) “mother-favored” discordance based on mother received PNC and her newborn did not; and (4) “newborn-favored” discordance based on mother did not receive PNC but the newborn did. We analyzed the association between the type of concordance/discordance and a set of socio-economic, demographic and health service variables, selected based on their potential relationship with childbirth (see **Box 1**). Given PNC is measured differently whether delivery occurred in a health facility or not, we also ran a stratified analysis by place of delivery. All covariates were self-reported by the women during the individual interview. Antenatal care content quality was defined as whether the woman reported having had blood pressure taken, and a blood and urine sample drawn at least once during ANC visits, and whether she took any iron supplementation during her pregnancy. Missing or “Don’t Know” responses were considered as “No”. We further stratified all analyses by DHS phases given differences in the way the PNC questions were asked. The DHS6 and DHS7 data were then pooled and a stratified analysis by place of delivery was carried out, given differences in the way PNC was measured by place of delivery. We report the proportions and 95% confidence intervals (CI) for all analyses.

Box 1 **Covariates included in the analysis.**

**Covariates: Socio-economic and demographic variables**

• Women reported level of education

• Marital status

• Maternal age at last birth

• Parity

• Wealth quintile

• Proceeding birth interval

• Whether the child is still alive

• Months since last birth

**Covariates: Service provision**

• Place of delivery (facility vs other)

• Cesarean delivery

• ANC content quality (having received blood pressure assessment, blood and urine test, and iron supplementation during the index pregnancy)

We assessed the correlates of PNC discordance/concordance by carrying out a multilevel logit regression of PNC discordance/concordance outcome defined as binary variable. The dichotomous outcome was discordance in PNC between the mother and newborn. The regressions were run on the pooled data and stratified by place of delivery, controlling for the type of discordance. We used Stata 14 with for all analysis (Stata Corp, College Station, TX, USA). For reporting proportions and 95% confidence intervals, we used the *svy* command in Stata, taking into account sampling weights, enumeration area clustering and stratified design. To account for differential sample size in the pooled data, the descriptive analysis was weighted by the inverse of the proportion of births in the past two years preceding the survey in each country.

### Ethical clearance

All data used are publicly available. Ethical clearance was the responsibility of the institutions that collected the data.

## RESULTS

### Reported timing of PNC

**Figure 3:**
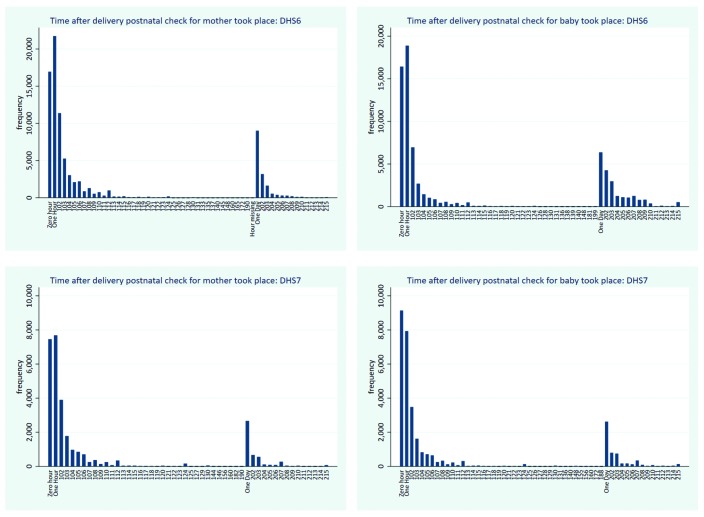
Timing of reported first PNC contact after delivery for mothers and newborn, by phase of Demographic and Health Survey (DHS).

**Figure 3** shows the timing of reported PNC contact for mothers and newborns by phase of the DHS. The distribution of PNC timing for mothers and for newborn appears similar across mothers and newborn and within each survey phase. The report of first PNC contact decreases rapidly beyond 1 hour; another peak is observed on day 1 followed with a decline in subsequent days.

### Sensitivity analysis of PNC coverage to reported timing

**Figure 4:**
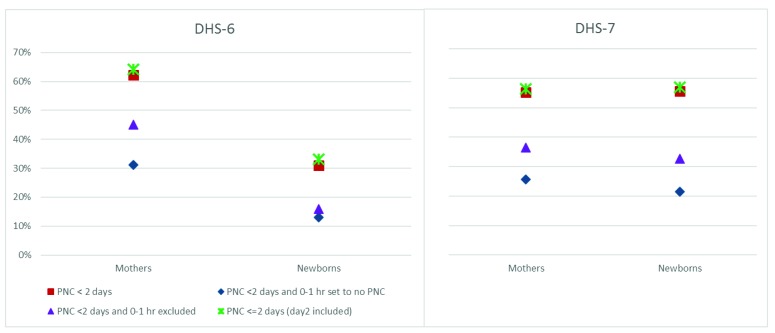
Sensitivity of postnatal care (PNC) indicators: comparison of PNC indicators for mothers and newborns, excluding/including 0-1 hours and/or day 2.

To capture the effects of the report of timing of the PNC contact on the overall PNC coverage indicator, **Figure 4** shows results of sensitivity analysis comparing the global PNC indicator (red square) to other variants computed separately for DHS 6 and 7 phases. These variants are: (1) PNC within 2 days, with any PNC reported to have occurred during 0 or 1 hour considered as no PNC (blue diamond); (2) PNC within two days with report of 0-1 hour timing excluded from the analysis (purple triangle); and (3) PNC within two days, including reported day 2 (green star). Further pooled and country specific results are included in Table S2, S3 and S4 in the **Online Supplementary Document**. For DHS6, coverage of PNC for mothers is substantially higher than that of babies, while for DHS7, the levels are similar. For DHS6, PNC reported to have occurred during the first 0-1 hour constitute almost half of PNC occurring within two days among women: PNC within two days was 62%, but when those reported to have occurred within 0-1 hour are considered as no PNC, the indicator drops to 31%. The drop is from 31% to 13% for newborn. Thus, for both mothers and babies, half of the report of PNC appears to have occurred within 0-1 hour of delivery. When PNC within 0-1 hour is excluded from the analysis, the drop reduces substantially for mothers only (from 62% to 45%). Similar results are observed for DHS7, although the drop between PNC within two days and PNC within two days with 0-1 set to no PNC was slightly more pronounced for babies (from 56% to 22%) than for mothers (55% to 26%).

### Gap between the Coverage of PNC for mothers and PNC for newborns

**Figure 5:**
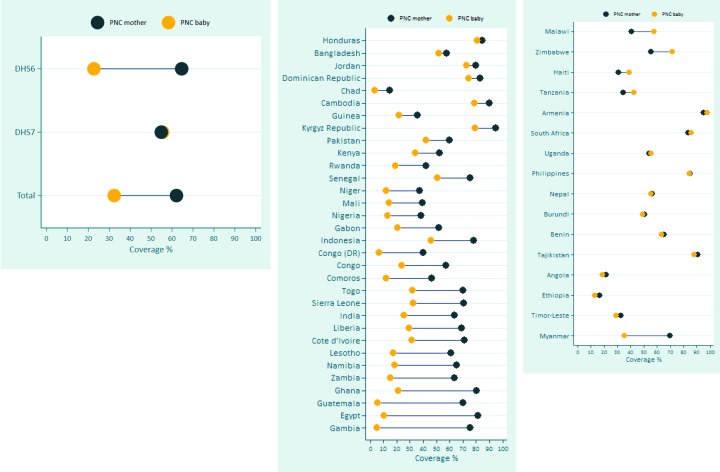
Gap in the coverage of postnatal care (PNC) for mothers and newborns. **Panel A.** Median gap across 48 countries. **Panel B.** Country specific gap, Demographic and Health Survey (DHS) 6 surveys. **Panel C.** Country specific gap, DHS 7 surveys.

There is a substantial gap in PNC coverage between mothers and babies in favor of mothers, especially for surveys of DHS6 (**Figure 5**). The overall gap in the median PNC across the 48 surveys is as large as 30 percentage points in favor of the mother (62% for mothers and 32% for babies), originating mostly from the gap in DHS 6 surveys, for which the median gap is as wide as 42 percentage points (65% for mothers and 23% for babies). For these surveys, the gap is over 50 percentage points (pp) in Gambia (71 pp), Egypt (71 pp), Guatemala (65 pp), and Ghana (59 pp) (**Figure 5**, Panel B). For DHS7, a few countries show large gap favoring the mother (Myanmar) or the newborn (Malawi, Zimbabwe), although the median PNC across the surveys is the same (55%) (**Figure 5**, Panel C).

### Level of discordance in PNC for mother and the newborn

**Figure 6:**
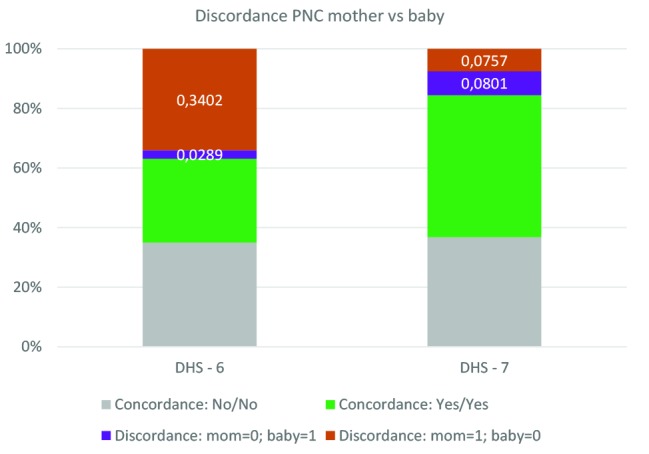
Discordance in postnatal care (PNC) for mother and newborn, pooled data across surveys by phase of Demographic and Health Survey (DHS).

**Figure 7:**
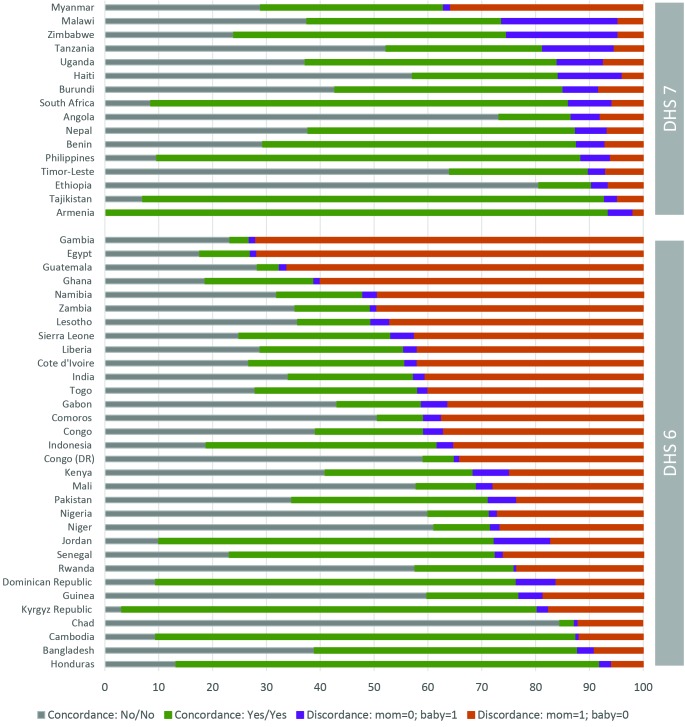
Discordance in postnatal care (PNC) for mother and newborn, by country and according to the survey phase.

**Figure 6** and **Figure 7** present the level of discordance/concordance between PNC mother and PNC newborn respectively in the pooled data across surveys stratified by DHS phase and by specific countries. Consistent with previous results, there is a substantially higher level of discordance in PNC between mother and babies from DHS 6 surveys than from DHS 7. Across the DHS 6 survey, discordance is observed in 37% of cases, and largely skewed in favor of the mother: 34% of mother received PNC while their babies did not, while the opposite occurred in only 3% of the cases. The total level of discordance across DHS 7 surveys is 16%, equally distributed between mothers and babies. Large variation in the level of discordance is observed across countries, ranging from 73% in Egypt and Gambia to 8% in Honduras for DHS 6 surveys, and from 37% in Myanmar to 7% in Armenia and Tajikistan. There are a few countries, especially with DHS 7, in which babies are favored in PNC than mothers. These includes Malawi (21% of mothers reported no PNC for themselves while the babies received one, compared 5% for the opposite), Zimbabwe (21% compared to 6%), Tanzania (13% compared to 6%), Haiti (12% compared to 4%), and Armenia (5% compared to 2%).

### Correlates of discordance in PNC

**Table 1** shows the percentage of mothers/babies by type of discordance/concordance according to socio-demographic characteristics and health service provision indicators. The table also shows 95% confidence intervals allowing comparison of the percentage across categories of the covariates. As shown above, level of discordance is largely in favor of the mother than the newborn and this is reflected across all characteristics considered. Thus, we focus on this type of discordance. A key set of results is related to service provision variables such the place of birth, receipt of antenatal care routine interventions, and delivery with cesarean section. Mothers who delivered in a health facility were largely more likely to report discordant PNC between themselves and their newborn than those who delivered outside a health facility who were more likely to report negative concordance. Among mothers who delivered in a health facility, 30% (95% CI = 29.7-30.6) had “mother-favored” discordance PNC compared to only 13% (95% CI = 12.3-13.3) among those who delivered outside facilities. This result appears to come mainly from DHS 6 surveys, where 44% of facilities deliveries had discordance compared to 19% among non-facility deliveries. Among DHS7, no such gap in discordance was observed between facility and non-facility deliveries (8% vs 7%, respectively) (Table S5 in the **Online Supplementary Document**). A related finding is when the mother reported receiving routine ANC interventions such as blood pressure assessment, blood and urine test, iron supplementation. Mothers who reported having received all these interventions during ANC were more likely to report higher level of mother-favored discordance (30% (95% CI = 29.8-30.9) vs 19% (95% CI = 18.9-19.7)). Furthermore, mothers who had caesarian section were more likely to report higher level of mother-favored (36% (95% CI = 35.0-37.1) vs 24% (95% CI = 23.2-24.0)).

**Table 1:** Percentage of mother/newborn by type of discordance/concordance with postnatal care (PNC) by socio-demographic and service provision characteristics

Characteristics	% Concordance No/No		% Concordance Yes/Yes		Discordance, Mom-Yes Baby-No		Discordance, Mom-No Baby-Yes		Total (%)	n	
**%**	**95% CI**	**%**	**95% CI**	**%**	**95% CI**	**%**	**95% CI**			
**Age at last birth (years), category:**											
<20	37.9	(37.0, 38.8)	31.5	(30.6,32.3)	25.2	(24.4, 26.0)	5.4	(5.1, 5.8)	100	41,712	
20-34	34.4	(33.9, 34.8)	36.1	(35.6,36.5)	25.0	(24.6, 25.4)	4.5	(4.3, 4.7)	100	202,047	
35+	39.4	(38.5, 40.3)	31.0	(30.1,32.0)	25.3	(24.5, 26.1)	4.3	(4.0, 4.7)	100	36,892	
**Months since last birth, category (age of child):**											
<6	35.9	(35.3, 36.6)	34.2	(33.5,34.9)	25.5	(24.9, 26.2)	4.4	(4.1, 4.6)	100	71,107	
6-11	35.1	(34.4, 35.7)	34.6	(34.0,35.3)	25.6	(25.0, 26.2)	4.7	(4.4, 5.0)	100	73,971	
12-23	35.6	(35.1, 36.2)	35.1	(34.5,35.6)	24.6	(24.1, 25.0)	4.7	(4.5, 4.9)	100	135,573	
**Parity, category:**											
1	29.0	(28.4, 29.6)	39.2	(38.5,39.8)	26.9	(26.3, 27.4)	5.0	(4.7, 5.3)	100	84,300	
2-3	32.3	(31.7, 32.9)	38.2	(37.6,38.9)	24.8	(24.3, 25.4)	4.6	(4.4, 4.9)	100	106,353	
4+	45.6	(44.9, 46.2)	26.4	(25.9,27.0)	23.7	(23.2, 24.3)	4.3	(4.0, 4.5)	100	89,998	
**Months of preceding birth interval, category:**											
<24	41.0	(40.0, 41.9)	32.7	(31.8,33.7)	22.0	(21.2, 22.8)	4.3	(4.0, 4.7)	100	34,669	
24-35	43.5	(42.8, 44.3)	28.1	(27.4,28.8)	24.3	(23.6, 24.9)	4.1	(3.9, 4.4)	100	61,023	
36+	35.0	(34.4, 35.6)	34.8	(34.3,35.4)	25.4	(24.9, 26.0)	4.7	(4.5, 4.9)	100	107,457	
First birth	27.7	(27.1, 28.3)	40.7	(40.0,41.4)	26.6	(26.0, 27.2)	5.0	(4.7, 5.3)	100	77,502	
**Wealth quintile:**											
Poorest	47.5	(46.7, 48.3)	27.9	(27.2,28.7)	20.2	(19.6, 20.8)	4.4	(4.1, 4.7)	100	63,327	
Poorer	40.4	(39.6, 41.2)	32.3	(31.5,33.1)	22.8	(22.1, 23.4)	4.6	(4.3, 4.9)	100	60,113	
Middle	34.9	(34.1, 35.7)	34.1	(33.2, 34.9)	26.4	(25.6, 27.1)	4.6	(4.3, 5.0)	100	57,153	
Richer	29.0	(28.2, 29.8)	37.7	(36.8, 38.7)	28.5	(27.7, 29.3)	4.8	(4.5, 5.2)	100	54,048	
Richest	21.4	(20.6, 22.2)	44.6	(43.5, 45.8)	29.2	(28.3, 30.2)	4.8	(4.4, 5.2)	100	46,009	
**Highest educational level**											
No education	53.0	(52.2, 53.8)	20.0	(19.5, 20.6)	23.8	(23.1, 24.5)	3.2	(3.0, 3.4)	100	76,899	
Primary	40.6	(39.9, 41.2)	30.4	(29.8, 31.1)	23.5	(22.9, 24.0)	5.5	(5.3, 5.8)	100	82,506	
Secondary +	21.1	(20.6, 21.6)	47.0	(46.3, 47.6)	27.0	(26.5, 27.6)	4.9	(4.7, 5.2)	100	121,247	
**Current marital status:**											
Never in union	30.0	(28.8, 31.2)	33.6	(32.2, 35.0)	30.9	(29.6, 32.3)	5.5	(4.9, 6.1)	100	20,106	
Married/living with partner	36.0	(35.5, 36.4)	34.8	(34.4, 35.3)	24.7	(24.4, 25.1)	4.5	(4.3, 4.6)	100	246,817	
Other	36.2	(34.7, 37.7)	35.0	(33.5, 36.6)	22.7	(21.4, 24.1)	6.0	(5.3, 6.9)	100	13,728	
**Facility delivery:**											
No	72.3	(71.6, 73.0)	11.1	(10.6, 11.5)	12.8	(12.3, 13.3)	3.8	(3.6, 4.1)	100	81,991	
Yes	20.4	(20.0, 20.8)	44.5	(44.0, 45.0)	30.2	(29.7,30.6)	5.0	(4.8, 5.1)	100	198,660	
**Last birth was with caesarean section:**											
No	38.7	(38.2, 39.1)	32.9	(32.5, 33.4)	23.6	(23.2, 24.0)	4.8	(4.7, 5.0)	100	247,316	
Yes	12.6	(11.9, 13.4)	48.1	(46.9, 49.3)	36.1	(35.0, 37.1)	3.2	(2.8, 3.7)	100	33,335	
**ANC: Blood pressure taken + Blood sample + Iron + Urine sample:**										
No	47.2	(46.6,47.8)	28.9	(28.3, 29.4)	19.3	(18.9, 19.7)	4.6	(4.4, 4.8)	100	133,913	
Yes	24.9	(24.5,25.4)	40.1	(39.5, 40.7)	30.4	(29.8, 30.9)	4.6	(4.4, 4.8)	100	146,738	
**Child is alive:**											
No	43.7	(41.9,45.4)	24.1	(22.6, 25.7)	27.2	(25.7, 28.8)	5.0	(4.2, 6.1)	100	9,656	
Yes	35.3	(34.9,35.7)	35.1	(34.7, 35.5)	25.0	(24.6, 25.4)	4.6	(4.5, 4.8)	100	270,995	

CI – confidence interval, ANC – antenatal care

There were also varying associations based on socio-demographic characteristics. While there is no difference in the level of discordance according to the age of the woman at the last birth or the child age at the time of survey, mothers with first birth and those with lower parity were more likely to report discordance than other women. Among mothers with parity one, 27% had mother-favored discordance compared to 25% among mothers with parity 2 or 3, and 24% among those with parity 4 or more. Birth interval is also associated with level of discordance, with shorter birth interval corresponding to lower level of discordance. Mothers with higher socio-economic status, those in higher wealth quintile or more educated, were more likely to report discordance than those with lower socio-economic status. Furthermore, mothers who were never in union were more likely to report discordant PNC than married/living together mothers or those of other marital status. Mothers of children who have died were more likely to report discordance (27% vs 25%).

**Table 2** shows the stratification of the analysis by place of birth, distinguishing facility births from non-facility births. The results are similar to those of all births presented in **Table 1** except for woman’s education level for which mothers with no education reported higher discordance compared to educated women.

**Table 2:** Percentage of mother/newborn by type of discordance with postnatal care (PNC) by socio-demographic and service provision characteristics according to the place of delivery

Characteristics	Health facility delivery						Non-health facility delivery					
**Discordance, Mom-Yes Baby-No**			**Discordance, Mom-No Baby-Yes**			**Discordance, Mom-Yes Baby-No**			**Discordance, Mom-No Baby-Yes**		
	**%**	**95% CI**		**%**	**95% CI**		**%**	**95% CI**		**%**	**95% CI**	
**Age at last birth, category:**												
<20	30.6	(29.6,	31.6)	6.1	(5.6,	6.6)	12.5	(11.5,	13.5)	3.9	(3.4,	4.5)
20-34	29.7	(29.2,	30.2)	4.8	(4.6,	5.0)	13.1	(12.5,	13.7)	3.8	(3.6,	4.1)
35+	32.2	(31.0,	33.3)	4.6	(4.1,	5.1)	12.0	(11.1,	13.0)	3.7	(3.2,	4.3)
**Months since last birth, category (age of child):**												
<6	30.8	(30.0,	31.6)	4.6	(4.3,	4.9)	12.8	(12.0,	13.7)	3.8	(3.3,	4.2)
6-11	30.7	(30.0,	31.5)	5.1	(4.7,	5.5)	12.9	(12.1,	13.7)	3.7	(3.3,	4.1)
12-23	29.5	(28.9,	30.1)	5.1	(4.8,	5.3)	12.8	(12.1,	13.4)	3.9	(3.6,	4.3)
**Parity, category**												
1	30.2	(29.6,	30.9)	5.1	(4.8,	5.5)	12.9	(12.1,	13.8)	4.4	(3.9,	5.0)
2-3	28.8	(28.2,	29.5)	4.9	(4.6,	5.2)	13.8	(13.1,	14.6)	3.9	(3.5,	4.3)
4+	32.1	(31.3,	32.8)	4.8	(4.5,	5.1)	12.0	(11.3,	12.7)	3.5	(3.2,	3.8)
**Months of preceding birth interval, category:**												
<24	28	(26.9,	29.1)	4.5	(4.0,	5.0)	11.2	(10.4,	12.2)	4.1	(3.6,	4.8)
24-35	31.9	(31.1,	32.8)	4.4	(4.1,	4.8)	12.5	(11.7,	13.3)	3.7	(3.3,	4.1)
36+	30.5	(29.8,	31.1)	5.2	(4.9,	5.5)	13.3	(12.6,	14.0)	3.6	(3.2,	3.9)
First birth	29.5	(28.8,	30.2)	5.2	(4.8,	5.5)	13.6	(12.7,	14.7)	4.3	(3.8,	5.0)
**Wealth quintile combined:**												
poorest	28.2	(27.3,	29.1)	5.3	(4.9,	5.8)	11.2	(10.5,	12.0)	3.3	(3.0,	3.7)
poorer	28.9	(28.0,	29.8)	5	(4.6,	5.4)	12.0	(11.3,	12.8)	3.8	(3.4,	4.3)
middle	31.3	(30.4,	32.2)	4.8	(4.5,	5.2)	14.1	(13.1,	15.1)	4.2	(3.7,	4.7)
richer	31.7	(30.7,	32.6)	5	(4.6,	5.4)	15.3	(14.1,	16.7)	4	(3.5,	4.7)
richest	30.2	(29.2,	31.3)	4.7	(4.3,	5.1)	17.6	(15.3,	20.1)	5.7	(4.6,	7.1)
**Highest educational level:**												
no education	37.1	(36.1,	38.1)	3.3	(3.1,	3.6)	11.8	[11.1,	12.5)	3	[2.7,	3.3)
primary	28.6	(27.9,	29.4)	6.1	(5.8,	6.5)	12.4	(11.7,	13.1)	4.3	(3.9,	4.8)
secondary +	28.6	(28.0,	29.2)	4.9	(4.6,	5.2)	16.3	(15.2,	17.4)	5.1	(4.6,	5.8)
**Current marital status:**												
Never in union	33.3	(31.8,	34.9)	5.6	(4.9,	6.4)	19.7	(17.4,	22.1)	4.9	(3.8,	6.1)
Married/living with partner	30.1	(29.6,	30.6)	4.8	(4.6,	5.0)	12.5	(12.0,	13.0)	3.7	(3.5,	4.0)
Other	26	(24.5,	27.7)	6.3	(5.4,	7.3)	12.9	(10.9,	15.4)	5.2	(4.1,	6.6)
**Last birth a caesarean section:**												
No	29.0	(28.5,	29.5)	5.3	(5.1,	5.5)	NA			NA		
Yes	36.1	(35.0,	37.1)	3.2	(2.8,	3.7)	NA			NA		
**ANC: BP taken + Blood sample + Iron + Urine sample:**												
No	26.2	(25.6,	26.8)	5.5	(5.2,	5.8)	9.9	(9.4,	10.4)	3.5	(3.2,	3.7)
Yes	32.7	(32.1,	33.3)	4.6	(4.4,	4.9)	19.4	(18.4,	20.5)	4.6	(4.2,	5.1)
**Child is alive:**												
No	34.3	(32.3,	36.4)	5.3	(4.1,	6.8)	14.7	(12.7,	16.8)	4.6	(3.6,	5.8)
Yes	30.0	(29.6,	30.5)	4.9	(4.8,	5.1)	12.7	(12.2,	13.3)	3.8	(3.6,	4.0)

CI – confidence interval, ANC – antenatal care, BP – blood pressure

**Table 3** presents odds ratios from multivariate logistic regression including all characteristics discussed above for all births and stratified by place of delivery. In the model with all births, delivery in a health facility and receipts of the four ANC interventions are significantly positively associated with PNC discordance (OR = 2.05 and 1.08 respectively). However, delivery with Caesarian section is no longer significant. As shown previously, DHS6 is more likely to show PNC discordance (OR = 1.89) that DHS7. The mother’s receipt of PNC is significantly associated with high discordance (OR = 52.3), while the opposite is observed for the newborn (OR = 0.02). Mothers are also likely to report discordant PNC for older newborn, those born 12-23 months before the survey. According to socio-demographic characteristics, mothers with high wealth or education are significantly more like to report discordant PNC. These results are generally maintained when the analysis is stratified by place of residence. For non-facility births, mothers whose children have died and those in other marital status category (divorce or widow) were more likely to report discordant PNC.

**Table 3:** Odds ratio of postnatal care (PNC) discordance between mother and newborn from multivariate logistic regression of all births in the two years preceding the survey, and by place of birth

Characteristics	All births, n = 280 651		Facility births, n = 198 012		Non-facility births, n = 82 639	
	**Odds ratio**	**95% CI**	**Odds ratio**	**95% CI**	**Odds ratio**	**95% CI**
**Age at birth (years):**						
<20	1.00		1.00		1.00	
20-35	0.98	(0.94, 1.02)	0.99	(0.94, 1.03)	0.97	(0.88, 1.06)
36+	0.95	(0.90, 1.01)	0.96	(0.90, 1.04)	0.94	(0.83, 1.06)
**Parity:**						
1 child	1.00		1.00		1.00	
2-3 children	1.01	(0.95, 1.06)	0.99	(0.93, 1.06)	0.98	(0.84, 1.14)
4+ children	1.00	(0.94, 1.06)	0.98	(0.91, 1.05)	0.95	(0.82, 1.11)
**Previous birth interval:**						
No previous births	1.00		1.00		1.00	
<24 months	0.99	(0.94, 1.04)	0.97	(0.92, 1.03)	1.09	(0.95, 1.26)
24-35 months	0.99	(0.95, 1.04)	0.99	(0.94, 1.04)	1.09	(0.95, 1.25)
36+ months	0.99	(0.95, 1.04)	0.99	(0.95, 1.05)	1.05	(0.91, 1.20)
**Wealth quintile:**						
Poorest	1.00		1.00		1.00	
2^nd^ poorest	1.04*	(1.01, 1.08)	1.03	(0.99, 1.08)	1.00	(0.94, 1.07)
3^rd^ poorest	1.10*	(1.06, 1.14)	1.06*	(1.01, 1.11)	1.08*	(1.00, 1.16)
4^th^ poorest	1.10*	(1.06, 1.15)	1.07*	(1.02, 1.12)	1.09	(1.00, 1.19)
Least poor	1.06*	(1.01, 1.11)	1.03	(0.98, 1.08)	1.12	(0.99, 1.27)
**Woman’s education:**						
No education	1.00		1.00		1.00	
Primary school	1.13*	(1.09, 1.17)	1.08*	(1.04, 1.13)	1.14*	(1.06, 1.22)
Secondary school+	1.13*	(1.09, 1.17)	1.09*	(1.04, 1.14)	1.16*	(1.08, 1.26)
**Woman’s marital status:**						
Married/living together	1.00		1.00		1.00	
Never in union	1.05	(0.98, 1.12)	1.04	(0.96, 1.12)	1.08	(0.93, 1.25)
Other (divorce, widow)	1.01	(0.95, 1.08)	0.95	(0.88, 1.02)	1.21*	(1.05, 1.39)
**Delivered in a facility delivery**	2.05*	(1.98, 2.12)	NA		NA	
**Survey type, DH6 (ref)**	1.89*	(1.65, 2.17)	2.22*	(1.90, 2.59)	1.24	(0.85, 1.81)
**Birth was c-section**	0.99	(0.95, 1.03)	1.04	(1.00, 1.08)	NA	
**Child is currently alive**	0.95	(0.89, 1.01)	0.98	(0.90, 1.06)	0.88*	(0.78, 0.99)
**Time interval between survey and the delivery (months):**						
<6	1.00		1.00		1.00	
6-11	1.03	(1.00, 1.06)	1.03	(0.99, 1.07)	0.99	(0.92, 1.07)
12-23	1.04*	(1.01, 1.07)	1.04*	(1.01, 1.08)	1.01	(0.95, 1.08)
**Received 4 ANC components during pregnancy**	1.08*	(1.05, 1.11)	1.06*	(1.03, 1.09)	1.15*	(1.08, 1.22)
**Mother received postnatal care**	52.26*	(50.64, 53.93)	46.93*	(45.26, 48.65)	50.16*	(47.13, 53.39)
**Baby received postnatal care**	0.02*	(0.02, 0.02)	0.01*	(0.01, 0.02)	0.22*	(0.20, 0.23)

CI – confidence interval, PNC – postnatal care, ANC – antenatal care

*P < 0.05. The logistic regressions controlled for specific country.

## DISCUSSION

The journey for a safe and healthy childbirth does not end with the delivery of a healthy newborn but continues during the postnatal period during which mother and newborn are still vulnerable to complications and infections that can lead to morbidity and death. The vulnerability of the postnatal period led the WHO to recommend sequences of health checks post-delivery for both the mother and the newborn, regardless of the place of birth, the first being within 24 hours. These health checks are different from care delivered to mothers and newborn during the intrapartum care, for which a separate set of recommendations exist [5].

While the recommendations for PNC appear straightforward, measuring and monitoring the coverage of such contacts continue to be a challenge in low and middle-income countries. These challenges lie in both the design and formulation of PNC questions asked in household surveys and women’s recall of these contacts and their timing as distinct from intrapartum care received during delivery in health facilities. Our analysis of 48 DHS implemented since 2011 and covering DHS phases 6 and 7 showed substantial measurement issues that are reflected in the reported coverage of PNC estimates for mothers and newborns. The development of PNC modules in DHS evolved over time both in terms of content of questionnaires and in the target population. We noticed substantial differences in the measurement of PNC for mother and newborn between phases 6 and 7. The content of the questionnaires for PNC newborn differed between DHS 6 and 7, with the later more clarified to improve the understanding of the questions. Such improvements have affected the coverage level of PNC newborn and reduced substantially the level of discordance between mother and newborn in the receipt of PNC. The large gap in the PNC coverage between mother and newborn (42 percentage points on average) and the high level of discordance between mother and newborn in the receipt of PNC (37%) observed in DHS 6 surveys almost disappeared in DHS 7 surveys. In DHS 6 surveys analyzed, PNC mother was estimated at 62% while that of the newborn was 23%. Subsequently, there were 37% discordant PNC between mother and newborn in phase 6, largely in favor of the mother (34%), while only 16% discordant cases were observed in DHS 7 surveys, equally distributed between mother and newborn. Thus, the observed substantial level of PNC discordance between mother and newborn appears driven more by measurement issues than by actual country programs not reaching newborns.

A sensitivity analysis of the reported timing of PNC for mother and newborn in regard to the global PNC indicators based on the first two days after delivery showed that over half of the PNC cases among mothers and newborns were reported to have occurred within the first hour of delivery. This is observed in both phases of the DHS and confirms the continued challenges in distinguishing intrapartum care which often continues up to an hour or more from PNC, and the accurate assessment of the two days following delivery. The reported levels of PNC coverage are therefore substantially overestimated. When PNC that was reported to have occurred within the one hour is considered as no PNC, the coverage levels dropped by half or more regardless of the DHS phase. For DHS 6 surveys analyzed, PNC mother dropped from 62% to 31% and PNC newborn dropped from 31% to 13%. Among DHS 7 surveys, it dropped respectively from 56% to 26% and from 57% to 22%. The size of drop reduced when the reported PNC cases within the first hour were excluded from the data set.

A further assessment of the level and correlates of discordance between PNC mother and newborn showed that women delivering in health facilities were more likely to report discordant PNC between mother and newborn. A total 35% of discordant cases were observed among facility deliveries compared to 16% for non-facility deliveries. This result is primarily because PNC level for non-facility deliveries is low in general. However, the level of discordance among health facility deliveries is concerning and is mostly observed in DHS phase 6 surveys (44% vs 16%) compared to DHS 7 surveys (8% vs 7%). This appears to be due to measurement artifact stemming from questionnaire design and implementation than to programs not reaching mothers and babies.

Our analysis showed that the past and current measurement of PNC for mother and newborn indicators in DHS include substantial measurement errors that challenge the validity of the reported coverage levels across countries, despite some improvements in questionnaires in the latest DHS phases. We have not included data from MICS in the analysis, but past reviews suggested that although coverage gap between mother and newborn is almost inexistent, the very high coverage generally reported may be affected by substantial overestimation due misclassification of intrapartum and immediate essential newborn care as PNC. MICS has adopted a detailed and more stable protocol for PNC measurement since around 2005 The DHS PNC module now follows a similar protocol as MICS but there are still some differences that may affected the comparability of the reported coverage levels.

Measurement errors in PNC contact also come from the recall interviews with mothers. Several studies have shown recall issues from mothers on maternal and newborn health care questions [9-11]. A qualitative study among women in Ghana revealed that mothers can only recall checks that were easily observed and for which the health provider explained the services being provided to the mothers. However, in most cases, mothers are not given any explanation about the care, and sometimes the newborn may be taken away into another room for care.[12] In Malawi and Bangladesh, women could not understand the health check questions, prompting DHS to revise the PNC questions to include example of health check. However, there remains a substantial confusion between intrapartum care and PNC visits [13]. Observation-based validation studies in Kenya and Swaziland have also demonstrated issues with women’s recall of care received during postnatal period [14,15].

The current global PNC trends databases based on DHS and MICS and other national surveys cannot be used to accurately capture trends in PNC coverage that are reflective of maternal and newborn health program and service delivery in countries. Further assessment and validation of measurement procedures are urgently warranted. Such initiatives must also consider the current gap in measuring the joint PNC of mother and newborn to support the desire to promote focus on both mother and newborn together.

## Additional material

Online Supplementary Document
