# Supplementary material for: Discordance in postnatal care between mothers and newborns: Measurement artifact or missed opportunity?
Source: J Glob Health. 2020 Mar 14;10(1):010505. doi: 10.7189/jogh.10.010505 (PMC7101084; doi:10.7189/jogh.10.010505)
Supplement: Online Supplementary Document [file jogh-10-010505-s001.pdf]

Table S1: Countries included in the analysis

County	Survey year	DHS Phase	No. of births in previous 24m (Most recent birth, unweighted)
Angola	2015	7	5,837
Armenia	2015	7	682
Bangladesh	2014	6	3,078
Benin	2017	7	5,486
Burundi	2016	7	5,261
Cambodia	2014	6	2,899
Chad	2014	6	6,590
Comoros	2012	6	1,255
Congo	2011	6	3,835
Congo (Democratic Republic of the)	2013	6	7,322
Cote d'Ivoire	2011	6	3,113
Dominican Republic	2013	6	1,430
Egypt	2014	6	6,410
Ethiopia	2016	7	4,081
Gabon	2012	6	2,511
Gambia	2013	6	3,481
Ghana	2014	6	2,329
Guatemala	2014	6	4,789
Guinea	2012	6	2,810
Haiti	2016	7	2,535
Honduras	2011	6	4,449
India	2015	6	97,935
Indonesia	2012	6	7,141
Jordan	2012	6	3,678
Kenya	2014	6	3,807
Kyrgyz Republic	2012	6	1,805
Lesotho	2014	6	1,387
Liberia	2013	6	3,064
Malawi	2015	7	6,538
Mali	2012	6	3,952
Myanmar	2015	7	1,863
Namibia	2013	6	2,051
Nepal	2016	7	1,970
Niger	2012	6	4,759
Nigeria	2013	6	12,397
Pakistan	2012	6	4,155
Philippines	2017	7	3,871
Rwanda	2014	6	3,169
Senegal	2015	6	5,353
Sierra Leone	2013	6	4,668
South Africa	2016	7	1,376
Tajikistan	2017	7	2,321
Tanzania	2015	7	4,134
Timor-Leste	2016	7	2,837
Togo	2013	6	2,782
Uganda	2016	7	5,992
Zambia	2013	6	5,096
Zimbabwe	2015	7	2,367

Table S2: Sensitivity analysis of the coverage of postnatal care for mother and newborn (pooled data by DHS phases)

Mother			Newborn		
**DHS 7**	**%**	**n**	**DHS 7**	**%**	**n**
Postnatal care for mothers, zero hour only	14.1%	57,151	Postnatal care for babies, zero hour only	18.0%	57,151
Postnatal care for mothers, 1 hr	15.5%	57,151	Postnatal care for babies, 1 hr	16.1%	57,151
Postnatal care for mothers, >1 hr post delivery	32.1%	57,151	Postnatal care for babies, >1 hr post delivery	32.3%	57,151
Postnatal care for mothers, don't know	1.5%	57,151	Postnatal care for babies, don't know	1.7%	57,151
Postnatal care for mothers <2 days	55.2%	57,151	Postnatal care for babies <2 days	55.7%	57,151
Postnatal care for mothers <2 days, (0-1 hours = no PNC)	25.6%	57,151	Postnatal care for babies <2 days, (0-1 hours = no PNC)	21.6%	57,151
Postnatal care for mothers <2 days, (0-1 hours excluded from indicator)	36.4%	40,249	Postnatal care for babies <2 days, (0-1 hours excluded from indicator)	32.7%	37,665
Postnatal care for mothers, day 2 only (code 202)	1.2%	57,151	Postnatal care for babies, day 2 only (code 202)	1.3%	57,151
Postnatal care for mothers <3 days	56.4%	57,151	Postnatal care for babies <3 days	57.0%	57,151
Postnatal care for mothers <3 days, (0-1hr= No PNC)	26.8%	57,151	Postnatal care for babies <3 days, (0-1hr= No PNC)	22.9%	57,151
Postnatal care for mothers <3 days, (0-1hr= excluded from indicator)	38.1%	40,249	Postnatal care for babies <3 days, (0-1hr= exclude)	34.7%	37,665
**DHS 6**			**DHS 6**		
Postnatal care for mothers, zero hour only	13.9%	223,500	Postnatal care for babies, zero hour only	9.6%	223,500
Postnatal care for mothers, 1 hr	17.1%	223,500	Postnatal care for babies, 1 hr	8.3%	223,500
Postnatal care for mothers, >1 hr post delivery	38.2%	223,500	Postnatal care for babies, >1 hr post delivery	39.2%	223,500
Postnatal care for mothers, don't know	1.8%	223,500	Postnatal care for babies, don't know	1.2%	223,500
Postnatal care for mothers <2 days	62.2%	223,500	Postnatal care for babies <2 days	31.0%	223,500
Postnatal care for mothers <2 days, (0-1 hours = no PNC)	31.2%	223,500	Postnatal care for babies <2 days, (0-1 hours = no PNC)	13.1%	223,500
Postnatal care for mothers <2 days, (0-1 hours excluded from indicator)	45.2%	154,245	Postnatal care for babies <2 days, (0-1 hours excluded from indicator)	16.0%	183,470
Postnatal care for mothers, day 2 only (code 202)	2.0%	223,500	Postnatal care for babies, day 2 only (code 202)	2.0%	223,500
Postnatal care for mothers <3 days	64.2%	223,500	Postnatal care for babies <3 days	33.1%	223,500
Postnatal care for mothers <3 days, (0-1hr= No PNC)	33.2%	223,500	Postnatal care for babies <3 days, (0-1hr= No PNC)	15.2%	223,500
Postnatal care for mothers <3 days, (0-1hr= exclude)	48.1%	154,245	Postnatal care for babies <3 days, (0-1hr= exclude)	18.5%	183,470
**DHS 6 & DHS 7 pooled**			**DHS 6 & DHS 7 pooled**		
Postnatal care for mothers, zero hour only	14.0%	280,651	Postnatal care for babies, zero hour only	12.4%	280,651
Postnatal care for mothers, 1 hr	16.5%	280,651	Postnatal care for babies, 1 hr	11.0%	280,651
Postnatal care for mothers, >1 hr post delivery	36.2%	280,651	Postnatal care for babies, >1 hr post delivery	36.8%	280,651
Postnatal care for mothers, don't know	1.7%	280,651	Postnatal care for babies, don't know	1.4%	280,651
Postnatal care for mothers <2 days	59.8%	280,651	Postnatal care for babies <2 days	39.4%	280,651
Postnatal care for mothers <2 days, (0-1 hours = no PNC)	29.3%	280,651	Postnatal care for babies <2 days, (0-1 hours = no PNC)	16.0%	280,651
Postnatal care for mothers <2 days, (0-1 hours excluded from indicator)	42.2%	195,026	Postnatal care for babies <2 days, (0-1 hours excluded from indicator)	20.9%	215,042
Postnatal care for mothers, day 2 only (code 202)	1.7%	280,651	Postnatal care for babies, day 2 only (code 202)	1.8%	280,651
Postnatal care for mothers <3 days	61.6%	280,651	Postnatal care for babies <3 days	41.1%	280,651
Postnatal care for mothers <3 days, (0-1hr= No PNC)	31.0%	280,651	Postnatal care for babies <3 days, (0-1hr= No PNC)	17.8%	280,651
Postnatal care for mothers <3 days, (0-1hr= exclude)	44.7%	195,026	Postnatal care for babies <3 days, (0-1hr= exclude)	23.2%	215,042

Table S3: Country specific sensitivity analysis of the coverage of PNC for mothers

County	Postnatal care for mothers, zero hour only	Postnatal care for mothers, 1 hr	Postnatal care for mothers, greater than 1 hr	Postnatal care for mothers, don't know	Postnatal care for mothers <2 days	Postnatal care for mothers <2 days, (0-1 hours = no PNC)	Postnatal care for mothers <2 days, (0-1 hours excluded from indicator)	Postnatal care for mothers, day 2 only (code 202)	Postnatal care for mothers <3 days	Postnatal care for mothers <3 days, (0-1hr= No PNC)	Postnatal care for mothers <3 days, (0-1hr= excluded from indicator)
Bangladesh	25.4	12.8	26.3	0.5	58.0	19.7	31.9	2.2	60.1	21.9	35.5
Cambodia	29.4	28.3	33.1	0.8	90.1	32.4	76.5	0.4	90.4	32.7	77.3
Chad	0.9	5.9	15.0	1.3	14.7	7.9	8.5	1.6	16.3	9.5	10.2
Comoros	2.8	13.8	38.6	9.9	46.2	29.6	35.5	2.9	49.0	32.5	38.9
Congo	6.2	8.9	59.3	3.6	57.2	42.0	49.5	7.0	64.1	49.0	57.7
Congo (Democratic Republic of the)	1.8	8.6	37.5	1.0	39.9	29.4	32.9	4.3	44.2	33.7	37.7
Cote d'Ivoire	19.9	23.3	35.6	1.3	71.0	27.9	49.0	1.8	72.8	29.7	52.2
Dominican Republic	12.2	21.3	59.8	2.6	83.2	49.7	74.8	1.2	84.5	51.0	76.6
Egypt	5.2	37.0	41.1	0.3	81.2	39.0	67.5	0.3	81.5	39.3	68.1
Gabon	4.6	9.5	52.5	4.6	52.0	37.8	44.1	7.6	59.6	45.5	53.0
Gambia	18.0	33.8	25.5	0.9	75.6	23.8	49.4	0.4	76.0	24.2	50.3
Ghana	8.0	28.0	49.5	0.4	80.2	44.1	69.0	1.7	81.8	45.8	71.6
Guatemala	0.1	3.9	82.8	0.1	70.2	66.3	69.0	7.4	77.7	73.7	76.7
Guinea	13.2	10.8	18.9	0.5	35.7	11.8	15.5	1.4	37.1	13.2	17.3
Honduras	3.0	12.0	75.3	0.5	84.6	69.6	81.8	0.4	84.9	69.9	82.3
India	14.5	29.3	25.7	0.7	63.8	19.9	35.5	1.6	65.4	21.6	38.4
Indonesia	15.0	23.4	49.4	1.5	78.1	39.7	64.4	2.4	80.4	42.1	68.3
Jordan	4.3	15.6	66.2	0.3	79.6	59.7	74.5	2.5	82.0	62.2	77.6
Kenya	13.1	12	33	0.3	52.3	27.2	36.4	1.5	53.8	28.7	38.3
Kyrgyz Republic	42.4	25.0	29.6	1.2	94.8	27.5	84.0	1.1	95.9	28.6	87.5
Lesotho	2.5	12.8	58.6	3.1	60.7	45.4	53.6	1.6	62.3	46.9	55.4
Liberia	8.9	26.9	37.8	2.0	68.8	33.0	51.3	2.1	70.8	35.0	54.6
Mali	8.5	14.9	22.1	2.7	39.2	15.7	20.5	0.8	40.0	16.6	21.6
Namibia	5.9	15.6	58.5	5.5	65.4	43.9	56.0	3.5	69.0	47.4	60.4
Niger	12.0	9.7	19.4	0.8	37.1	15.3	19.6	0.6	37.7	15.9	20.3
Nigeria	10.0	11.8	19.9	0.4	38.5	16.7	21.3	1.2	39.7	17.9	22.9
Pakistan	26.3	18.5	16.7	1.0	59.9	15.1	27.4	0.4	60.4	15.5	28.2
Rwanda	14.0	8.0	23.5	0.1	41.9	19.8	25.5	1.1	43.0	21.0	26.9
Senegal	51.2	8.7	20.4	3.8	75.4	15.4	38.6	0.7	76.1	16.1	40.3
Sierra Leone	21.2	22.5	33.9	3.3	70.7	27.1	48.1	2.5	73.2	29.6	52.5
Togo	23.2	19.3	37.7	1.1	70.1	27.6	48.0	1.6	71.7	29.2	50.8
Zambia	22.9	14.6	31.4	3.5	63.5	26.0	41.5	0.4	63.9	26.4	42.2
Angola	3.0	6.8	27.5	2.2	21.5	11.6	12.9	2.1	23.6	13.8	15.3
Armenia	16.5	18.1	63.6	0.4	95.2	60.6	92.7	2.2	97.4	62.8	96.0
Benin	21.2	18.2	27.9	6.4	65.4	25.9	42.9	0.7	66.1	26.6	44.0
Burundi	16.3	12.5	23.5	0.0	50.7	21.9	30.7	0.4	51.1	22.3	31.3
Ethiopia	3.2	5.6	10.7	0.2	16.4	7.6	8.3	0.2	16.6	7.8	8.6
Haiti	6.1	6.7	27.3	0.1	31.0	18.2	20.8	2.4	33.3	20.6	23.6
Malawi	12.2	9.2	27.3	1.8	40.9	19.5	24.8	1.6	42.4	21.0	26.8
Myanmar	19.2	16.7	40.9	0.3	69.8	33.9	52.8	1.5	71.3	35.4	55.3
Nepal	17.3	16.7	24.4	0.4	56.4	22.4	33.9	0.4	56.7	22.8	34.5

Philippines	18.8	23.8	47.5	0.9	84.9	42.4	73.8	1.2	86.1	43.5	75.8
South Africa	37.0	22.9	28.1	6.1	83.4	23.5	58.6	0.3	83.7	23.8	59.4
Tajikistan	13.4	42.2	38.0	1.0	90.6	35.0	78.8	1.2	91.8	36.2	81.6
Tanzania	4.8	8.7	24.3	0.5	34.5	21.0	24.3	0.9	35.4	21.9	25.4
Timor-Leste	2.8	11.7	36.6	1.1	32.8	18.2	21.3	1.8	34.6	20.0	23.5
Uganda	16.9	13.5	26.5	1.1	54.2	23.8	34.2	0.7	54.9	24.5	35.3
Zimbabwe	16.7	12.8	41.9	1.6	55.5	26.0	36.9	1.4	56.9	27.5	38.9

Table S4: Country specific sensitivity analysis of the coverage of PNC for newborns

County	Postnatal care for babies, zero hour only	Postnatal care for babies, 1 hr	Postnatal care for babies, greater than 1 hr	Postnatal care for babies, don't know	Postnatal care for babies <2 days	Postnatal care for babies <2 days, (0-1 hours = no PNC)	Postnatal care for babies <2 days, (0-1 hours excluded from indicator)	Postnatal care for babies, day 2 only (code 202)	Postnatal care for babies <3 days	Postnatal care for babies <3 days, (0-1hr= No PNC)	Postnatal care for babies <3 days, (0-1hr= excluded from indicator)
Bangladesh	26.3	11.7	25.3	0.4	52.0	14.0	22.5	2.6	54.6	16.5	26.7
Cambodia	26.4	26.8	26.2	1.1	78.7	25.5	54.5	0.2	78.9	25.6	54.8
Chad	0.2	1.1	14.2	0.7	3.4	2.2	2.2	1.1	4.5	3.3	3.3
Comoros	1.5	4.8	20.6	6.7	11.9	5.6	6.0	1.8	13.7	7.4	7.9
Congo	2.7	5.4	46.6	0.9	23.8	15.7	17.1	4.3	28.1	20.0	21.8
Congo (Democratic Republic of the)	0.5	1.8	14.9	0.1	6.8	4.5	4.6	1.4	8.2	5.9	6.0
Cote d'Ivoire	7.8	10.3	49.5	0.5	31.3	13.2	16.2	3.2	34.5	16.4	20.0
Dominican Republic	25.0	22.4	44.0	1.8	74.3	26.9	51.2	2.2	76.6	29.1	55.5
Egypt	0.8	3.1	32.5	0.2	10.6	6.8	7.1	3.7	14.3	10.5	10.9
Gabon	3.3	3.8	46.7	0.9	20.7	13.6	14.6	4.8	25.5	18.4	19.8
Gambia	0.9	0.9	26.4	0.1	4.8	3.0	3.0	1.0	5.7	4.0	4.0
Ghana	1.3	6.5	64.0	0.1	21.4	13.6	14.8	1.5	22.9	15.2	16.4
Guatemala	0.0	0.2	86.2	0.8	5.5	5.3	5.3	2.6	8.1	7.9	7.9
Guinea	4.1	6.6	45.7	0.3	21.5	10.8	12.1	3.6	25.2	14.4	16.2
Honduras	7.0	15.7	68.5	3.0	80.9	58.2	75.3	0.4	81.3	58.6	75.8
India	7.2	10.6	18.9	0.2	25.4	7.6	9.3	1.9	27.3	9.5	11.5
Indonesia	11.7	15.2	37.2	2.3	46.0	19.2	26.2	2.0	48.0	21.1	28.9
Jordan	13.1	21.8	55.7	2.5	72.8	37.9	58.2	1.9	74.7	39.8	61.2
Kenya	10.2	9	44.5	0.4	34.2	15	18.6	1.4	35.7	16.5	20.4
Kyrgyz Republic	43.3	20.1	24.5	1.4	79.3	15.9	43.4	0.6	79.8	16.5	44.9
Lesotho	1.6	3.1	73.3	1.6	17.2	12.5	13.1	1.2	18.4	13.7	14.3
Liberia	3.5	9.9	48.2	0.6	29.1	15.7	18.1	5.5	34.6	21.2	24.4
Mali	4.2	5.6	24.6	1.7	14.3	4.6	5.1	1.3	15.6	5.9	6.5
Namibia	2.8	5.2	40.2	2.6	18.6	10.7	11.6	1.2	19.9	11.9	12.9
Niger	4.8	3.1	51.5	0.5	12.3	4.4	4.8	0.7	13.0	5.1	5.6
Nigeria	2.7	4.6	19.3	0.3	12.9	5.6	6.1	1.2	14.1	6.8	7.3
Pakistan	24.8	10.9	15.3	0.8	41.9	6.2	9.6	1.0	42.9	7.2	11.2
Rwanda	9.5	2.9	13.1	0.1	18.9	6.5	7.4	0.4	19.3	6.9	7.9
Senegal	40.9	2.4	34.8	3.6	50.9	7.5	13.3	0.8	51.6	8.3	14.6
Sierra Leone	7.4	9.6	49.3	1.5	32.6	15.7	18.9	6.4	39.1	22.1	26.7
Togo	8.6	7.9	52.9	0.5	32.1	15.6	18.7	3.0	35.2	18.6	22.3
Zambia	5.0	4.0	45.6	0.4	15.2	6.1	6.8	0.7	15.9	6.8	7.5
Angola	3.1	7.8	28.5	1.8	18.8	7.9	8.9	2.1	20.8	10.0	11.2
Armenia	30.4	25.9	42.0	1.5	97.9	41.5	95.1	0.3	98.2	41.9	95.9
Benin	22.4	17.3	27.7	6.0	63.6	23.9	39.6	0.9	64.5	24.8	41.1
Burundi	18.0	12.3	21.4	0.0	49.0	18.8	26.9	0.5	49.5	19.2	27.6
Ethiopia	2.7	4.8	9.8	0.3	12.9	5.4	5.8	0.3	13.2	5.7	6.2
Haiti	13.3	8.0	52.0	0.1	39.0	17.8	22.6	3.9	42.9	21.7	27.6
Malawi	20.4	13.0	37.6	2.4	57.8	24.4	36.7	2.0	59.8	26.4	39.7
Myanmar	10.8	8.3	25.7	0.4	35.4	16.2	20.1	1.0	36.4	17.3	21.4

Nepal	20.5	16.5	28.6	0.9	55.6	18.7	29.6	1.1	56.8	19.8	31.4
Philippines	23.1	24.9	45.2	1.6	84.3	36.3	69.8	1.4	85.7	37.7	72.5
South Africa	48.6	23.9	18.5	5.8	85.7	13.2	48.1	0.5	86.2	13.7	50.0
Tajikistan	14.6	42.0	36.3	1.7	88.2	31.6	72.8	1.3	89.6	32.9	75.9
Tanzania	8.5	11.9	34.7	0.8	42.3	21.9	27.5	1.0	43.4	22.9	28.8
Timor-Leste	2.2	10.7	35.9	1.1	29.1	16.2	18.6	1.7	30.7	17.9	20.5
Uganda	17.5	14.2	30.1	1.0	55.4	23.7	34.6	0.8	56.1	24.4	35.8
Zimbabwe	30.2	14.3	43.6	1.7	71.4	26.9	48.5	2.1	73.5	29.0	52.3

Table S5: Percentage of mother/newborn by type of discordance/concordance with PNC by socio-demographic and service provision characteristics, according the phase of the DHS survey

	DHS 7						DHS 6					
	% Concordance No/No	% Concordance Yes/Yes	Discordance Mom - Yes Baby - No	Discordance Mom - No Baby - Yes	Total (%)	n	% Concordance No/No	% Concordance Yes/Yes	Discordance Mom - Yes Baby - No	Discordance Mom - No Baby - Yes	Total (%)	n
	%	%	%	%			%	%	%	%		
**Age at last birth, category**												
<20	39.8	43.1	6.6	10.5	100.0	7,780	37.1	26.2	33.5	3.2	100	34,643
20-34	35.2	49.5	7.6	7.7	100.0	41,825	34.0	29.1	34.2	2.8	100	159,546
35+	42.4	42.0	8.6	6.9	100.0	7,547	37.8	25.4	33.9	3.0	100	29,311
**Parity, category**												
1	27.8	55.2	8.0	9.0	100.0	16,057	29.6	31.8	35.5	3.1	100	69,346
2-3	33.1	51.5	7.6	7.8	100.0	23,419	31.9	30.6	34.8	2.8	100	81,183
4+	49.8	35.7	7.1	7.4	100.0	17,676	43.5	22.0	31.7	2.8	100	72,971
**Preceding birth interval, category**												
<24	43.2	43.7	6.9	6.1	100.0	7,497	39.7	26.6	30.4	3.3	100	26,738
24-35	47.2	39.2	6.3	7.2	100.0	11,605	41.8	22.9	32.6	2.7	100	50,236
36+	35.7	47.9	8.1	8.4	100.0	21,827	34.7	28.2	34.3	2.8	100	85,685
First birth	27.8	55.2	8.1	8.9	100.0	16,223	27.6	33.0	36.4	2.9	100	60,841
**wealth index combined**												
poorest	48.4	37.9	6.1	7.6	100.0	12,932	47.0	22.8	27.4	2.7	100	50,359
poorer	41.8	43.7	6.9	7.6	100.0	12,365	39.7	26.3	31.0	3.0	100	47,625
middle	36.7	47.3	8.0	7.9	100.0	11,604	34.0	27.3	35.7	3.0	100	45,584
richer	30.9	52.1	8.2	8.7	100.0	10,875	28.0	30.5	38.7	2.8	100	43,303
richest	21.0	61.5	9.2	8.4	100.0	9,375	21.6	36.0	39.4	3.0	100	36,629
**highest educational level**												
no education	60.6	28.0	6.5	5.0	100.0	11,759	50.4	17.4	29.7	2.5	100	69,019
primary	46.7	34.8	8.3	10.3	100.0	18,116	37.1	28.0	32.1	2.8	100	63,083
secondary +	20.0	64.7	7.5	7.8	100.0	27,277	21.8	36.4	38.6	3.2	100	91,398
**current marital status**												
Never in union	27.5	56.0	6.2	10.3	100.0	4,320	31.4	21.2	44.6	2.8	100	15,561
Married/living with partner	37.2	47.4	7.7	7.7	100.0	50,081	35.3	28.4	33.4	2.9	100	196,916
Other	43.0	38.5	8.0	10.4	100.0	2,750	32.9	33.3	30.0	3.8	100	11,023
**Facility delivery**												
No	78.6	9.9	7.2	4.3	100.0	16,427	69.2	11.6	15.6	3.6	100	65,832
Yes	19.9	62.9	7.7	9.5	100.0	40,724	20.7	35.0	41.7	2.6	100	157,668
**last birth a caesarean section**												
No	39.5	45.4	6.8	8.4	100.0	51,799	38.2	26.3	32.6	2.9	100	194,089
Yes	10.7	69.4	15.2	4.7	100.0	5,352	13.3	40.4	43.6	2.7	100	29,412

**child is alive**												
no	46.8	33.3	11.5	8.3	100.0	1,820	42.2	19.9	34.4	3.5	100	7,982
yes	36.4	48.1	7.4	8.0	100.0	55,331	34.7	28.4	34.0	2.9	100	215,518
**Months since last birth, category (age of child)**												
<6	37.4	47.3	7.8	7.6	100.0	14,655	35.2	27.3	34.8	2.7	100	56,278
6-11	36.1	48.1	7.6	8.2	100.0	14,667	34.6	28.1	34.4	2.9	100	59,699
12-23	36.8	47.6	7.4	8.1	100.0	27,829	35.0	28.6	33.4	3.0	100	107,523
**ANC: BP taken + Blood sample + Iron + Urine sample**												
Did not get all 4 components	47.4	37.5	7.1	8.0	100.0	29,766	47.1	23.8	26.4	2.7	100	101,664
Got all 4 components	25.2	58.6	8.1	8.0	100.0	27,385	24.8	31.7	40.4	3.1	100	121,836
